# Book Review

**Published:** 1916-07

**Authors:** 


**Encyclopædia Medica.** Second Edition. Vol. III., Chloroform to Dyspnœa. Pp. 672. Edinburgh and London: W. Green & Son. 1916. Price 20s.—Another volume of "Green," the larger articles of which have been written and now revised by some forty contributing authorities in their respective subjects, under the general editorship of Dr. J. W. Ballantyne, who is the author of four of the major articles, is before us. The first article, on chloroform, commences with an historical introduction by the late Sir Alexander R. Simpson, and we note that the author, Dr. McAllum, calls attention to the fallacies of the corneal reflex, the common method being useless as a test of surgical anæsthesia. The well-known excellence of the articles in this encyclopædia is well maintained, but a detailed review of each volume is unnecessary.

**Modern Medicine and some Modern Remedies.** By THOMAS BODLEY SCOTT. Pp. xi., 159. London: H. K. Lewis. 1916.—These practical notes for the general practitioner are published with a preface by Sir Lauder Brunton, Bart., F.R.S., who writes that "it is a most welcome occurrence when a man fully qualified to do so writes down the ripe experiences of his life so as to help his fellow-workers, both general practitioners and consultants, who one and all may learn from him." The author apologises for "this *horæ subsecivæ* of a busy doctor whose ideas are not many, nor are they consecutive," but he is an experienced physician with special privileges, having a son who is an expert in bacteriology and the manufacture of vaccines, whilst the father ponders over and speculates on the results. In Chapter iii., "Therapeutic Speculations and Doubts," he gives a résumé of our present-day knowledge of ductless gland therapeutics, and makes many practical suggestions on their inter-association and possible uses. Much is being learnt on these endocrine organs, and the knowledge obtained is producing most important results, which have not yet come into general therapeutic use, but "with this new knowledge of the *vis medicatrix*, of its mechanism and of its chemistry, we must realise that our control over disease is enormously increased, and that there is a far brighter and less suffering future for the sons of men." Many useful suggestions are given in Chapter i., which is an excellent summary of all recent knowledge in "Disorders of the Heart," a chapter which requires to be read and pondered over before we can assimilate its valuable lessons. It serves to concentrate most of the more modern knowledge of heart affections into more practical and useful methods of management and therapeutics. The same remark applies to the chapter on "Arterio-sclerosis," and the final one on "Chronic Bronchitis and Bronchial Asthma," for both of which the practice of organic therapeutics seems likely to do much good.

**Index-Catalogue of the Library of the Surgeon-General's Office, United States Army.** Second Series. Vol. XX. V.—Water Works. Pp. 595. Washington: Government Printing Office. 1915.—The present volume is smaller than its predecessors, yet it includes 4,566 author titles, representing 2,263 volumes and 3,517 pamphlets, and also contains 4,151 subject titles of separate books and pamphlets, and 22,977 titles of articles in periodicals. The subjects of "Water" and "Waters" occupy no less than 164 pages of closely-written references. It is a perennial pleasure for us to record our high appreciation of the labours of those concerned in the compilation of this most indispensable work.

**The Medical Annual, 1916.** Bristol: John Wright & Sons Ltd.—The thirty-fourth volume of this Year-Book of Treatment and Practitioner's Index has contributions from twenty-eight contributors, and maintains its usual characters, with the addition of much new matter relating to questions of naval and military surgery. Deputy Surg.-General A. Gascoign Wildey, R.N., gives much valuable information in a general review on "The Medical Officer and the Fighting-man," and Dr. A. C. Inman gives some twenty-five pages on "Wound Infections, their Bacteriology, Biology and Treatment." The editors express their deep regret that, after eight years of collaboration in the production of the *Annual*, they can no longer obtain the assistance of the late Sir Charles Bent Ball, Bart. The special injuries to organs and nerves are considered under their proper headings in the body of the work, and it will be seen that great changes of views have taken place, especially in regard to the surgical treatment of wounds.

In a note by Dr. Graham Little on Ringworm we find that the condition long known by Hebra's name, Eczema Marginatum, or Eczematoid Ringworm, has been differentiated by Sabouraud from the parasites of ringworm by cultural characteristics, and by the clinical observation that this parasite never attacks the hair. The fungus is found solely in the superficial layer of the epidermis, and Sabouraud called it the Epidermophyton Inguinale. It may usually be successfully treated by Whitfield's Ointment, containing 3 per cent. of salicylic acid and 5 per cent. of benzoic acid. In more obstinate cases a chrysarobin ointment, although somewhat irritating, is more effective, but it is of importance that the clothing which may have come into contact with the disease should be thoroughly disinfected.

